# Whole grain foods and health – a Scandinavian perspective

**DOI:** 10.3402/fnr.v57i0.18503

**Published:** 2013-02-12

**Authors:** Wenche Frølich, Per Åman, Inge Tetens

**Affiliations:** 1 Norwegian School of Hotel Management, University of Stavanger, Jar, Norway; 2 Department of Food Science, Swedish University of Agricultural Sciences (SLU), Uppsala, Sweden; 3 Division of Nutrition, National Food Institute, Technical University of Denmark, Søborg, Denmark

**Keywords:** whole grain, nutrients, phytochemicals, processing, health effects

## Abstract

The food-based dietary guidelines in the Scandinavian countries that recommend an intake of minimum 75 g whole grain per 10 MJ (2,388 kcal) per day are mainly derived from prospective cohort studies where quantitative but little qualitative details are available on whole grain products. The objective of the current paper is to clarify possible differences in nutritional and health effects of the types of whole grain grown and consumed in the Scandinavian countries. A further objective is to substantiate how processing may influence the nutritional value and potential health effects of different whole grains and whole grain foods. The most commonly consumed whole grain cereals in the Scandinavian countries are wheat, rye, and oats with a considerable inter-country variation in the consumption patterns and with barley constituting only a minor role. The chemical composition of these different whole grains and thus the whole grain products consumed vary considerably with regard to the content of macro- and micronutrients and bioactive components. A considerable amount of scientific substantiation shows that processing methods of the whole grains are important for the physiological and health effects of the final whole grain products. Future research should consider the specific properties of each cereal and its processing methods to further identify the uniqueness and health potentials of whole grain products. This would enable the authorities to provide more specific food-based dietary guidelines in relation to whole grain to the benefit of both the food industry and the consumer.

In the Scandinavian countries (Norway, Sweden, and Denmark) food and health authorities recommend an increased consumption of whole grain cereals as part of their food-based dietary guidelines (1–3). The guidelines recommend an intake of at least 75 g whole grain per 10 MJ (2,400 kcal) and are based on the available scientific evidence that suggests that an increased consumption of whole grain may be associated with a reduced risk of several chronic diseases. The ‘Keyhole Symbol’ has been introduced as a helping aid for the consumers to eat more healthy (4). The focus in this guideline is on reduction of fat, sugar, and salt and increase of dietary fiber and whole grain foods. Every food group has its own criteria as to the level of certain nutrients to guide the consumers for the better choice.

In the Scandinavian countries whole grain is defined to include all parts of the naked cereal seed (caryopsis) from wheat (including spelt), rye, oats, barley, maize, rice, millet, and sorghum (5, 6). The whole grain can be consumed as intact, ground, cracked, or flaked caryopsis whose principle anatomical components – the starchy endosperm, germ, and bran – are present in the same relative proportions as they exist in the intact caryopsis. This definition is similar to the definition established by the American Association of Cereal Chemist (AACC) International. One important difference though is that the latter definition includes pseudocereals like amaranth, buckwheat, and quinoa that are not included in the Scandinavian definition.

It is important that a clear distinction is made between whole grain and whole grain foods. The concept of whole grain refers to the whole grain itself, whereas whole grain foods are foods containing a defined amount of whole grain cereals. The criteria for bread and other cereal foods to be labeled whole grain foods include a minimum content of whole grain calculated on a dry matter basis. To be allowed to use the ‘Keyhole Symbol’ in the Scandinavian countries, soft bread should contain at least 25% whole grain, and crisp bread, pasta, breakfast cereals, and porridge should contain at least 50% whole grain to fulfill the requirements. For flours, flakes, and kernels the content of whole grain should be 100% in the three countries. Thus, there is an important distinction between the content of nutrients in whole grain and whole grain foods, which also varies with the type of whole grain.

Naked cereal grains have basically the same anatomical structure but important differences appear in the chemical composition, which may also affect their nutritional value. Processing of the cereals can be of either dry or wet nature, depending on the type of grain and the products to be prepared. This may affect in various ways the chemical and nutritional composition as well as physical structures and functional properties. Generally, these differences are not taken into account when it comes to formulating dietary guidelines.

The scientific evidence for the potential health-promoting role of whole grains is mainly derived from prospective cohort studies where little is known about the source of whole grain and hardly anything about processing of the whole grain. Most of the prospective cohort studies reported in the literature are from large American cohorts where the dominant whole grain cereal is wheat. It is interesting to note that in these American studies corn consumed as popcorn is an important source of whole grain.

Among the Scandinavian countries, Sweden is the only country that has data on whole grain intake in their most recent national dietary survey among adults (18–80 years) (7). The Swedish survey showed an average intake of 39 and 46 g of whole grain/d for females (*n*=1,005) and males (*n*=792), respectively. In Denmark, data from the Danish National Survey of Dietary Habits and Physical Activity 2000–2004 were used to estimate the whole grain intake among children (4–14 years) and adults (15–75 years). The results showed an average intake among the children (*n*=1,159) of 28 g/d, among the females (*n*=2,503) of 28 g/d, and among the males (*n*=2,189) of 39 g/d (5). Rye bread was the major single source of whole grain intake with a contribution to the total intake comprising 58 and 64% for the children and adults, respectively.

A recently published descriptive study of a subgroup of the Scandinavian ‘HELGA’ population (*N*=8,702), based on dietary data collected from a 24 h dietary recall in 1995–2000, showed distinct national differences in the sources of whole grain intake (8). In the populations studied whole grain rye made up more than 70% of the total whole grain intake in Denmark, more than 50% in Sweden, but only 20% in Norway. Wheat was the main contributor of whole grain among Norwegian women. The study also showed that whole grain oats constituted, on average, between 6 and 19% of the total whole grain intake in the three countries. The potential contribution to health from whole grain of different cereals has until now been given little attention. Thus, the potential importance of the specific nutrient content and bioactive compounds found in the different whole grain cereals may not yet have been fully acknowledged (9). Even less consideration has been given to the potential effects of different processing methods used to prepare whole grain foods. Recently the European Food Safety Authority (EFSA) considered that whole grain is not sufficiently characterized in relation to claimed health effects (10).

The objective of this article is to clarify possible differences in nutrition and health effects of the types of whole grain grown and consumed in the Scandinavian countries. A further objective is to substantiate how processing may influence the nutritional value of whole grain and whole grain foods.

## Whole grain and health

A consistent inverse association between intake of whole grain foods and cardiovascular diseases (CVD) was reported in a meta-analysis of prospective cohort studies where six out of the seven prospective cohort studies were on US cohorts (11). The results of the meta-analysis suggested that an average 2.5 servings/day vs. 0.2 servings/day was associated with a 21% lower risk of CVD events in adult populations. This result is similar to an earlier meta-analysis by Anderson et al. (12).

An inverse association was also found between intake of whole grain foods and risk of type 2 diabetes in a systematic review based on data from six large US cohort studies (13). The results suggested that a two-serving-per-day increment in whole grain consumption was associated with a 21% decrease in the risk of type 2 diabetes after adjustment for potential confounders and BMI. Similar results were obtained in the Nurses’ Health Studies I and II that included more than 160,000 women (13).

In a systematic review and analysis of 15 observational studies on whole grain consumption and measures of body weight and adiposity, Harland and Garton (14) found that an increased consumption of three servings of whole grain foods per day was associated with a reduction in BMI of 0.630 kg/m^2^ and in waist circumference of 2.7 cm. Sub-group analyses showed no differences in the effects between genders or between locations (11 data pairs from the US and 8 from Europe).

Whole grain foods have also been associated with a reduced risk of some cancers and the evidence from prospective studies the associations are strongest for cancer in the gastrointestinal tract. A recent meta-analysis concluded that an increment of three servings daily of whole grain foods was associated with a reduction in risk of colorectal cancer of between 11 and 17% (15). Whole grain cereal consumption has also been associated with other gastrointestinal health benefits, including the prebiotic and the laxation effects (16).

The mechanisms for this protective associations between intake of whole grain foods and reduced risk of certain chronic diseases are diverse – and to a large extent unknown (17, 9). Research suggests that the protective effects of whole grain foods are due to the synergetic effects of the different types of dietary fibers and a multiple number of micronutrients and phytochemicals present as compared with refined grains (18). While the exact nature of the positive effects is still unknown, it is well recognized that different whole grain cereal foods contain different amounts and composition of micronutrients (like vitamins and minerals), dietary fiber, and phytochemicals (19).

Until recently, the number of whole grain intervention studies has been relatively small, and most have been conducted in at-risk populations and with small numbers of subjects and various sources of whole grain foods (20). Evidence from these intervention studies is variable (21).

In conclusion, the current scientific evidences from prospective cohort studies and from epidemiological observational studies suggest convincingly that whole grain plays an important role in reducing the risk of CVD and type 2 diabetes and that whole grain foods may play a protective role in body weight management, certain types of cancer, and gastrointestinal health. The lack of consistency in the results obtained from randomized intervention trials strongly suggests that other factors than those commonly studied are of importance for the nutritional/health effects. These others factors could, for example, be type of whole grain and processing methods.

## Types of whole grain

The most commonly consumed whole grain cereals in the Scandinavian countries are wheat, rye, and oats. Barley has mainly been used in animal feed as well as for production of malt and only to a limited extent for human consumption. Wheat and rye are naked cereals (caryopsis), which means that the husk has fallen off during threshing in the field, whereas oats and barley are generally covered cereals, which have to be dehulled during the milling process. After dehulling, the oat groats are classified as whole grain. The same should be true for barley if all components of the caryopsis could be retained after pearling.

Today most of the wheat and rye is consumed as sifted flour with variable extraction rates in the different Scandinavian countries. Norway has the highest extraction rate of sifted wheat at about 80%, and the extraction rate of this type of flour is 76–80% in Denmark and 70–72% in Sweden. The higher extraction rate the higher proportion of dietary fiber and associated compounds will be included in the refined cereal foods. In population studies, it is important to consider this fact when comparing health effects after intake of refined cereal products vs. whole grain foods. In Denmark, there are two different extraction rates for sifted rye flour, 88 and 80%. The extraction rate for this type of flour in Sweden is also 80%, while Norway has the lowest extraction rate for sifted rye flour with 75%.

The gross composition of whole grain wheat, rye, oats, and barley, mostly grown in the Scandinavian countries, differs between the cereals (Table 1). The values given in the National Food Composition tables for the different cereals are rather similar in the three countries and are therefore here given as a mean. Wheat and oats (oat flakes) are high in protein compared to rye and especially barley (pearled barley flakes). Wheat and barley have the highest content of starch, whereas rye is high in dietary fiber and sugars and oats is high in fat. However, large variations exist in the composition depending on cultivar and growing conditions.

**Table 1:** Gross chemical composition of whole grain wheat, whole grain rye, oat flakes (whole grain) and pearled barley flakes (per 100 g as eaten)

Component	Wheat	Rye	Oats	Barley
Protein	11.2	8.9	12.7	9.0
Starch	59.9^a^	54.0^c^	62.1^b^	62.1^a^
Sugars	1.25	3.7	1.4	0.8
Fat	2.3	2.4	7.1	2.2
Ash	1.5^c^	1.6^c^	2.1^c^	1.2^c^
Total DF	11.4	14.4	10.3	10.3

a Data from Sweden not included.

b Data from Sweden and Denmark not included.

c Data from Norway not included.

Figures are mean values from the Official National Food Composition Tables in Norway (22), Denmark (23), and Sweden (24).

The newly accepted definition for dietary fiber in Europe (Commission directive 2008/100/EC) includes all types of resistant starch as well as resistant oligosaccharides. With this definition, whole grain rye contains as much as 20% dietary fiber on a dry matter basis, whereas whole grain oats, analyzed as dehulled oats, contain only 10% (Table 2). Wheat contains approximately 13% and naked barley 15% dietary fiber. In the older food composition tables, the dietary fiber component fructan (including fructooligosaccharides) is essentially not included in the figures for total dietary fiber and therefore significantly lower figures are generally seen, especially for rye which has a high content of fructooligosaccharides (about 4%).

**Table 2:** Dietary fibre content and composition of in whole grain wheat, whole grain rye, dehulled oats, and naked barley.^a^

Component	Wheat	Rye	Oats	Barley
Total DF	13.5	19.9	10.2	15.2
Arabinoxylan	5.6	8.9	2.0	5.2
Cellulose	2.5	2.9	1.3	1.9
*β*-Glucan	0.8	1.5	5.0	4.6
Fructan	1.3	4.1	0.2	1.6
Klason lignin	0.8	1.1	1.4	0.7

Results given as % of dry matter.

a Dietary fibre analyzed as components included in the Uppsala method (AOAC 994.13) and fructan method (AOAC 999.03). *β*-Glucan analysed by AOAC method 32–23. Arabinoxylan and cellulose calculated as described by Andesson et al. (25). Results for wheat published by Andersson et al. (26) and for rye by Andersson et al. (25). Results for oats and barley are from unpublished analyses in P. Åman's laboratory.

Cereals’ fibers can be classified into two groups: one (wheat and rye) containing starchy endosperm cell walls where arabinoxylan dominates (about 75% of cell walls) and the other (oats and barley) where *β*-glucans are dominating (about 75% of the cell walls) (27). Rye has a higher content of arabinoxylan compared to wheat, depending on a higher proportion of cell walls in the starchy endosperm, and oats have a considerably lower content than wheat. Cellulose is mainly present in the outer parts of the caryopsis together with arabinoxylan and Klason lignin. The content of cellulose is low in dehulled oats and barley, and higher in wheat and rye. Dehulled oats and barley are high in *β*-glucan, while rye has an intermediate content and wheat a low content. Thus, it is obvious that the content and composition of dietary fiber in the different Scandinavian whole grain cereals differ significantly.

The different dietary fiber components have been shown to give different physiological effects (28). Insoluble and less fermentable dietary fiber components (lignified cell walls with arabinoxylan and cellulose as main components) in the outer parts of the caryopsis will influence passage rate and give high bulking effects in the large intestine. Arabinoxylan and especially *β*-glucan in the starchy endosperm are partly extractable viscous dietary fiber components and may influence the rate of absorption of nutrients in the small intestine and reabsorption of bile acids. Fructan, which has lower molecular weight than the other dietary fiber components in cereals, is highly extractable and fermentable and will thus generally not influence the absorption of other nutrients in the small intestine to any notable extent. It is thus evident that the physiological effects of dietary fiber differ depending on which cereal has been consumed and thereby the nutritional and potential health effect.

From the Official National Food Composition tables in the three countries, the content of certain vitamins in the four whole grain cereals seems to be in the same range (Table 3). However, the content of niacin seems to be considerably higher in whole grain wheat and barley compared to whole grain rye and oats. Also, the content of vitamin E in barley seems to be lower than in the other whole grains.

**Table 3:** Content of certain vitamins and minerals in whole grain of wheat, whole grain rye and oat flakes (whole grain) and pearled barley flakes (per 100 g as eaten).

Content	Wheat	Rye	Oats	Barley
Vitamin E (mg)	1.2	1.0	0.8	0.4
Thiamine (mg)	0.4	0.4	0.5	0.2
Riboflavin (mg)	0.1	0.2	0.1	0.1
Niacin (mg)	4.1	1.2	1.6	4.5
Vitamin B6 (mg)	0.3	0.3	0.2	0.3
Folate (µg)	35	48	45	25
Phosphorous (mg)	331	348	451	272
Iron (mg)	4.2	3.4	4.3	2.7
Calcium (mg)	31	29	50	25
Potassium (mg)	393	403	394	328
Magnesium (mg)	121	96	122	66
Selenium (µg)	5.8	2.8	1.0	2.1
Zinc (mg)	2.6	2.4	2.9	1.4
Copper (mg)	0.3^a^	0.3^a^	0.3^a^	0.4^a^

a Data from Sweden not included.

Figures are mean values from the Official National Food Composition Tables in Norway (22), Denmark (23), and Sweden (24).

For some minerals, a somewhat higher content can be found in whole grain oats compared to the other cereals (Table 3). The content of phosphorous present as phytic acid is also higher in oats than in other cereals. Most probably the rest of the minerals present in oats is associated with the phytic acid. It is interesting to note that average content of selenium in whole grain wheat is higher than in the other cereals. The explanation for this is most probably due to the Norwegian import of American wheat with a high content of selenium.

The content of some selected bioactive components varies greatly between the four whole grain cereals (Table 4). Both cultivar and growing conditions will also have an influence on the content, dependant on the bioactive component (37). No single variety could be selected as having the highest overall level of bioactive components or being more stable across environments. The nature of the positive physiological effects exerted by whole grain cereals remains unresolved due to this multitude of components and their interactions (9). Many of these components such as phenolic acids, flavanoids, alkylresorcinols, phytic acid, and phytosterols have numerous physiological functions and recognized health benefits. However, the synergy between the actions of these components is poorly characterized. It seems in certain cases that the dietary fiber components act as a carrier of the bioactive components (38). The transportation of the dietary bioactive components through the gastrointestinal tract has therefore been suggested to be an essential function of dietary fiber.

**Table 4:** Content of major bioactive components of whole grain of wheat, whole grain rye, oats, and barley (µg per g dry matter)

Component	Wheat	Rye	Oats^a^	Barley^a^
Phytic acid^b^	390–1,350	540–1,460	420–1,160	380–1,116
Tocols	28–80^c^	44–67^d^	16–36^e^	46–69^f^
Phenolic acids	326–1,171^g^	491–1,082^d^	351–873^e^	254–675^f^
Phytosterols	670–960^g^	1,098–1,420^d^	618–682^e^	899–1,153^f^
Alkylresorcinols	220–650^h^	797–1,231^d^	Not present	32–103^f^
Avenantramides	Not present	Not present	42–91^e^	Not present

a Husked and naked cultivar.

b Schlemmer et al. (29).

c Lampi et al. (30).

d Nyström et al. (31).

e Shewry et al. (32).

f Andersson et al. (33).

g Li et al. (34); Nurmi et al. (35)

h Andersson et al. (36).

## Importance of processing

The importance of the structure of foods was already pointed out for fruits by Harber and co-workers (39) who showed that an intact apple had higher satiety scores and a lowering effect on plasma glucose and serum insulin than apple puree and fiber-free apple juice with the fiber added. The structure of cereal foods has long been recognized as a parameter governing the health benefits of whole grain foods. The importance of the structure of cereals was pointed out by Holt and Miller (40). They studied meals comprising whole grains, cracked grains, and course and fine whole grain wheat flour and found that the smaller the particle size of the food, the higher was the glycaemic-insulin response and the lower the satiety rating. In a recently published randomized test meal study including healthy subjects, the postprandial glucose and appetite measures were compared after consumption of whole grain vs. refined wheat bread and pasta (41). The results showed no differences in postprandial glucose responses to the whole grain vs. refined wheat bread, whereas the pasta meals resulted in significantly lower glucose responses compared with the bread meals. Whole grain wheat bread but not whole grain wheat pasta reduced appetite measures compared to refined wheat bread. These studies indicate that the complexity and the interaction between cereal source and processing need to be unraveled.

Later studies have shown that preserving the natural initial fibrous network, especially in more or less intact wheat, rye, oats, and barley kernels, seems to be of importance for the physiological responses like satiety and glucose metabolism. Also, formed compact structures like in pasta and dumplings can give positive responses on the same parameters (42). It has also been shown that the particle size of the milled whole grain is of importance for the bulking effects in the large intestine (43, 44).

Amount, molecular weight distribution, structure, and conformation of fermentable dietary fiber are also of importance for the physiological effects in the intestine, such as blood glucose attenuation and serum cholesterol lowering properties. The solubility of the dietary fiber varies greatly between whole grain cereals and is as high as 37% for rye but lower for the other cereals (25). The extractability of dietary fiber will also be influenced by the processing conditions; for example, components can be released or degraded by endogenous and/or added fiber degrading enzymes or become insoluble by aggregation (45). It is well known that the molecular weight of extractable dietary fiber components can be highly affected by different processing conditions. During wet processing, such as baking, endogenous *β*-glucan degrading enzymes can reduce the molecular weight of the polymer. This will lead to changed properties such as reduced viscosity and modified gelling properties, which may be of importance for the physiological responses. Similar modifications may take place with extractable arabinoxylan, but this polymer is more resistant to degradation/fermentation than *β*-glucan due to a more complex structure requiring several enzymatic activities. It has been shown in a human study that a reduced molecular weight of the *β*-glucan gives a reduced cholesterol lowering effect (46). A scientific opinion by EFSA also states that oat *β*-glucan may be degraded during purification and manufacturing of foods, affecting considerably its physiochemical properties (47). Consequently, the cholesterol lowering effect of oat *β*-glucan may be weakened or may even disappear during processing.

Resistant starch is defined as the starch that is not digested and absorbed in the small intestine and is therefore being classified as dietary fiber. Resistant starch is present naturally in some foods with intact botanical structures, like in intact barley and rye kernels, but can also be formed during heat treatment. During milling or homogenization of the food, the intact botanical structures could be opened; thereby, the content of resistant starch will be lowered. During hydrothermal processing, resistant starch can be formed due to recrystallization of amylose, resulting in a higher dietary fiber content in the cereal foods. The content will, however, vary due to processing conditions. It has, for example, been shown that preparation of whole grain rye flour porridge will increase the content of resistant starch with as much as 3% units compared to the raw ingredients (48). Resistant starch is a source of fermentable carbohydrate for the large bowel micro flora and appears to favor the butyrate production (49).

Whole grain cereals, especially rye, can be a rich source of fructan. In whole grain rye around 4% is present. The content of fructan may, however, decrease during bread making. Hansen et al. followed the fructan content during rye bread making and observed a 26% decrease in freshly prepared dough, 35% after proofing, and 45% in the crumb after oven baking (50). Sour dough baking reduced the fructan content up to 62% (25).

Whole grain foods are valuable sources of minerals. A high content of phytate in these products has been considered a factor for limited bioavailability of these nutrients. Degradation of phytate may, however, result in an increased bioavailability of the minerals (29). This could be done during food processing like soaking, germination, malting, and fermentation. At optimal conditions for the enzyme phytase (55°C, pH 4.5–5.0) the phytate could be effectively reduced after 12–16 h of soaking. The acidity of the dough during breadmaking is of great importance for phytate degradation during scalding and sour dough fermentation. After 8 h of fermentation at 37°C, a reduction of 65% of the phytate content may be obtained in regular dough, compared to 97% in sour dough.

Unprocessed oats show similar phytase activity as wheat (51). Due to the high fat content in oats, unprocessed oats are heat treated to avoid rancidity during storage and preparation. During this treatment, the naturally occurring enzymes, including phytase, will be totally inactivated. As oats do not contain gluten, this cereal is not used as the only type of flour when making doughs for large-loaf volume breads. When oats are mixed with other flours, the phytate in oats could be degraded by the phytase from other flours present in the bread dough as phytase is not art specific for the different cereals (29). However, in products made from solely oats, e.g. porridge, the minerals in the oats may have a limited bioavailability.

## Conclusions

Food and health authorities in the Scandinavian countries recommend an increase in the consumption of whole grain to a minimum of 75 g whole grain per 10 MJ (2,388 kcal) per day with little specification on the type of whole grain to be consumed. The different chemical composition and physical properties together with the a variety of processing methods in the preparation of the whole grain foods consumed in the Scandinavian countries may to an extent explain the different nutrition and health outcomes observed in different types of studies. Future research should consider the specific properties of each cereal and their processing methods to further identify the uniqueness and health potentials of whole grain products. This would enable the authorities to provide more specific food-based dietary guidelines in relation to whole grain to the benefit of both the food industry and the consumer.
